# The *Paeonia qiui* R2R3-MYB Transcription Factor PqMYB113 Positively Regulates Anthocyanin Accumulation in *Arabidopsis thaliana* and Tobacco

**DOI:** 10.3389/fpls.2021.810990

**Published:** 2022-01-12

**Authors:** Xiaokun Liu, Jingjing Duan, Dan Huo, Qinqin Li, Qiaoyun Wang, Yanlong Zhang, Lixin Niu, Jianrang Luo

**Affiliations:** ^1^College of Landscape Architecture and Arts, Northwest A&F University, Xianyang, China; ^2^National Engineering Research Center for Oil Peony, Yangling, China; ^3^Shanghai Key Laboratory of Plant Functional Genomics and Resource, Shanghai, China; ^4^College of Plant Science, Tarim University, Alar, China

**Keywords:** *Paeonia qiui*, anthocyanin, transcription factor, MYB, positive regulation

## Abstract

*Paeonia qiui* is a wild species of tree peony native to China. Its leaves are purplish red from the bud germination to the flowering stage, and anthocyanin is the main pigment in purplish red leaves. However, the anthocyanin synthesis regulation mechanism in tree peony leaves remains unclear. In this study, an R2R3-MYB, PqMYB113 was identified from the leaves of *P. qiui*. Phylogenetic analysis revealed that PqMYB113 clustered with *Liquidambar* LfMYB113 and grape VvMYBA6. Subcellular location analysis showed that PqMYB113 was located in the cell nucleus. The transient reporter assay suggested that PqMYB113 was a transcriptional activator. The overexpression of *PqMYB113* in *Arabidopsis thaliana* and tobacco (*Nicotiana tabacum*) resulted in increased anthocyanin accumulation and the upregulation of *CHS*, *F3H*, *F3’H*, *DFR*, and *ANS*. The dual luciferase reporter assay showed that PqMYB113 could activate the promoters of *PqDFR* and *PqANS*. Bimolecular fluorescence complementation assays and yeast two-hybrid assays suggested that PqMYB113 could form a ternary MBW complex with PqbHLH1 and PqWD40 cofactors. These results provide insight into the regulation of anthocyanin biosynthesis in tree peony leaves.

## Introduction

Anthocyanins are widely distributed in plant flowers, fruits and other tissues (Jaakola, 2013). It provides plants with red, orange, purple, blue, and other different colors that attract pollinators and/or make plants resist abiotic and biotic stresses such as ultraviolet light, cold, drought and microbial agents (Shang et al., 2011; Song et al., 2016; Jin and Zhu, 2019; An et al., 2020). Over the past few decades, as a branch of the flavonoid pathway, anthocyanin biosynthesis has been extensively studied in *Arabidopsis*, grape, apple, and many other plants (Tanaka et al., 2008; Jaakola, 2013). It is well known that anthocyanin biosynthesis is controlled by a series of structural genes (enzyme genes), including the chalcone synthase gene (*CHS*), chalcone isomerase gene (*CHI*), flavanone 3-hydroxylase gene (*F3H*), flavonoid 3′-hydroxylase gene (*F3*′*H*), flavonoid 3′,5′-hydroxylation gene (*F3*′*5*′*H*), dihydroflavonol 4-reductase gene (*DFR*), anthocyanin synthase gene (*ANS*), etc (Tanaka et al., 2008).

In addition to these structural genes, anthocyanin biosynthesis is also regulated by transcription factors, mainly myeloblastosis (MYB), basic helix loop helix (bHLH) and WD40/WDR (Allan et al., 2008; Hichri et al., 2011; Li, 2014). Among them, MYB plays a pivotal role in regulating anthocyanin synthesis (Dubos et al., 2010). It can act alone or with bHLH and WD40 constitutes the MYB-bHLH-WD40 complex (MBW) to regulate the structural genes of the anthocyanin pathway (Jaakola, 2013; Li, 2014; Xu et al., 2015). The MYB transcription factor family is divided into 4 categories according to the number of N-terminal conserved domains, namely, one conserved domain 1R (R1/2, R3-MYB), two conserved domains 2R (R2R3-MYB), three conserved domains 3R (R1R2R3-MYB) and four conserved domains 4R (Dubos et al., 2010). Among these four types of MYB transcription factors, R2R3-MYB is the most important in anthocyanin synthesis (Liu et al., 2015). Many R2R3-MYB transcription factors that positively regulate anthocyanin biosynthesis have been identified from various plants, such as AtMYB75 (PAP1), AtMYB90 (PAP2), AtMYB113, and AtMYB114 in *Arabidopsis* (Stracke et al., 2007; Gonzalez et al., 2008), PhAN2 and PhAN4 in *Petunia hybrid* (Albert et al., 2011), MdMYB1, MdMYBA, MdMYB10, and MdMYB110a in apple (Takos et al., 2006; Ban et al., 2007; Espley et al., 2007; Chagne et al., 2013), PpMYB10.1, PpMYB10.2, and PpMYB10.4 in peach (Rahim et al., 2014; Zhou et al., 2016). In addition to these activators, some MYB repressors were also found, such as FaMYB1 in *Fragaria* × *ananass*a (Paolocci et al., 2011), VvMYBC2-L1/L2/L3 and VvMYB4-like in *Vitis vinifera* (Cavallini et al., 2015), PtrMYB182 in *Populus spp*. (Yoshida et al., 2015) and PpMYB18 in *Prunus persica* (Hui et al., 2019). However, most of these studies focus on anthocyanin biosynthesis regulation in flowers or fruits, and the molecular mechanism of red color formation in leaves is still not well known.

As a wild tree peony native to China, *P. qiui* is a typical spring-red leaved plant that has purple–red leaves from the bud germination to the flowering stage, which has good foliage value. Based on our transcriptome sequencing, forty MYB genes were identified from differentially expressed genes. Among them, two R2R3-MYBs that may be related to anthocyanin biosynthesis were screened (Luo et al., 2017). One was designated as PqMYB4, its inhibitory function in anthocyanin biosynthesis was confirmed by our team (Huo et al., 2020). Another R2R3-MYB (Unigene0024459) showed highest consistency with LfMYB113 which regulated anthocyanin biosynthesis in *Liquidambar formosana*, so it was designated as PqMYB113 (Luo et al., 2017). In this study, PqMYB113 was isolated from *P. qiui* leaves, and the expression level of *PqMYB113* gene was consistent with the change in anthocyanin content in leaves. The stable transformation in *Arabidopsis* and tobacco showed that PqMYB113 could promote anthocyanin accumulation. The dual luciferase reporter assay showed that PqMYB113 could activate the promoters of *PqDFR* and *PqANS*. Bimolecular fluorescence complementation assays suggested that PqMYB113 could form a ternary MBW complex with PqbHLH1 and PqWD40. This study provided a basis for revealing the molecular mechanism of foliage color change in *P. qiui* and genetic resources for the molecular breeding of tree peony with colored leaves.

## Materials and Methods

### Plant Materials

The *Paeonia qiui* plants used in this study were grown under field conditions at Northwest A&F University, Yangling, Shaanxi, China (altitude, 432 m above sea; 34°15′N, 108°3′E). Eight-year-old tree peonies were planted in loam, and the soil moisture content was approximately 17.5%. The second young leaves from the top of plants were collected in 10, 20, 30, 40, and 50 days after sprouting and designated as S1, S2, S3, S4, and S5, respectively. The samples were collected in the morning during March and April 2021 (10°C–18°C in the day and 5°C–8°C in the night). *Arabidopsis* and tobacco were cultivated in a climate chamber (22°C/80%; 16 h light and 8 h darkness). All samples were immediately frozen in liquid nitrogen and then stored at −80°C until use.

### Total RNA Extraction and Quantitative Real-Time Polymerase Chain Reaction

Total RNA was isolated from the different leaf stages of *P. qiui* using the TIANGEN RNA Prep Pure Plant kit (Tiangen Biotech Co., Ltd., Beijing, China). The quality and concentration of RNA samples were tested by Goldview-stained agarose gel electrophoresis and spectrophotometric analysis, respectively. According to the manufacturer’s instructions of the PrimeScript^®^ RT reagent Kit (DRR047A, Takara, Japan), 1 μg DNA-free RNA sample was used to synthesize cDNA and stored at −20°C. qRT-PCR was conducted using SYBR^®^ Premix Ex Taq™ II (DRR041A, Takara, Japan), and ubiquitin was used as an internal control (Luo et al., 2017). The primer sequences used for qRT-PCR are listed in Supplementary Table 1. The relative expression level of the qRT-PCR assay was calculated with the 2^–ΔΔ*CT*^ method, and a dissolution curve was automatically generated by software. Three biological replicates were performed.

### Isolation of the Full-Length Coding Sequence of PqMYB113 and Bioinformatics Analysis

The gene specific primers *PqMYB113-F* and *PqMYB113-R* were designed by Oligo 7.0 software (Supplementary Table 1), and full-length gene amplification was carried out using the cDNA of *P. qiui* leaves as a template according to TransTaq-T DNA Polymerase. The specific bands of the expected size PCR products were recycled by the agarose gel DNA extraction kit (Takara, Japan). Then, the expected products were ligated into the pMD19-T vector placed at 4°C overnight, and the ligation products were transformed into *Escherichia coli* competent cells. After positive screening identification, the correct bacterial solution was sent to the company for sequencing. The open reading frame of *PqMYB113* was found by the ORF Finder online tool in NCBI^1^. A homology search of sequences was carried out through BLAST in NCBI, and multiple alignments were analyzed using DNAMAN 8.0. A phylogenetic tree was constructed using the neighbor-joining method (NJ) with MEGA 7.0 software.

### Subcellular Localization

The ORF of *PqMYB113* without the stop codon was inserted into the pCAMBIA1301-GFP vector. Then, the constructed plasmid was bombarded into onion epidermal cells using a Biolistic PDS1000 instrument (Bio-Rad, CA, United States). After incubation at 25°C for at least 16 h in the dark, the samples were observed under a confocal laser scanning microscope.

### Overexpression Vector Construct and *Arabidopsis* and Tobacco Transformation

The full-length cDNA of *PqMYB113* was inserted in the sites between *Kpn*I and *Sal*I of the pCAMBIA1300 vector. Then, the recombinant vector was introduced into *Agrobacterium tumefaciens* strain GV3101 for *Arabidopsis* and tobacco transformation. pCAMBIA1300-PqMYB113 in *Agrobacterium* strain GV3101 was transformed into wild-type *Arabidopsis* plants using the floral dip method. *Agrobacterium*-mediated transformation of tobacco plants was subsequently carried out. Hygromycin (200 mg/L) was used to select the transgenic tobacco lines.

### Dual Luciferase Transient Transfection Assay and Dual Luciferase Reporter Assay

For the dual luciferase transient transfection assay, p35S-GAL4-BD, the effector plasmid, the reporter plasmid containing firefly luciferase and internal plasmids containing *Renilla* luciferase were prepared as described previously (Zong et al., 2016). For the effector plasmid, AtMY75 (positive control), AtMYBL2 (negative control) and PqMYB113 cDNA fragments were inserted into the *Eco*RI site of p35S-GAL4-BD plasmids. The effector, reporter and internal plasmids were delivered into *Arabidopsis* protoplasts by PEG-mediated transformation of protoplasts. The relative luciferase (LUC/REN) activity was assayed with the Dual-Luciferase Reporter^®^ Assay System (Promega) using a Promega GloMax 20/20 Microplate luminometer.

For the dual luciferase reporter assay, the promoters of *PqDFR* and *PqANS* were amplified using the Genome Walking Kit and inserted into the vector pGreenII 0800-LUC with the primers listed in Supplementary Table 2. PqMYB113 was joined with the pGreenII 62-SK vector, which was driven by 35S promoter. The primers used for reporter and effector constructions were listed in Supplementary Table 3. All of these recombinant plasmids were individually transformed into *Agrobacterium* strain GV3101. Activity data were expressed as the ratio of LUC activity to REN activity. Blank controls were run with only the promoter-LUC reporter construct (no transcription factor).

### Bimolecular Fluorescence Complementation Experiment

*PqMYB113* without the stop codon was constructed between the two restriction sites (*Bam*HI and *Xhol*) of the pSPYNE-35S expression vector. *PqbHLH*1 and *PqWD40* without the stop codon were constructed into *Bam*HI and *Xhol* of the pSPYCE-35S expression vector, respectively. In other words, PqMYB113 was connected to the N-terminus of YFP, and PqbHLH1 and PqWD40 were fused to the C-terminus of YFP. The constructed vector was transformed into *Agrobacterium* strain GV3101 and infiltrated into tobacco leaves. After 72 h of co-infiltration, samples were taken to detect the fluorescence signal by confocal laser scanning microscope.

### Yeast Two Hybrid Assay

PqMYB113, PqbHLH1, and PqWD40 were constructed into the pGADT7 vector and pGBKT7 vector, respectively, and then co-transfected into yeast AH109. The medium lacking Leu and Trp (–T/–L) was used for selecting the transformed yeast strains at 30°C for 3 days. Subsequently, putative transformants were transferred to medium lacking Trp, Leu, His and adenine (–T/–L/–H/–A) with or without X-α-Gal, and cultured for 2–4 days. pGADT7-T and pGBKT7-Lam or pGADT7-T and pGBKT7-53 were co-transformed as negative and positive controls.

### Anthocyanin Content Measurement

Ten milligrams of seedlings were ground in liquid nitrogen. Extraction of anthocyanins was performed in 250 μL of methanol containing 1% HCl in the dark at 4°C for 24 h. Then chlorophyll was eliminated by adding 250 μL chloroform and 250 μL water. Subsequently, the samples were centrifuged for 10 min at 10,000 × *g* at 4°C. The supernatant was used for anthocyanin measurement using a spectrophotometer at 530 and 657 nm. The relative anthocyanin level was calculated by the formula (A530-0.33 × A657)/mg.

## Results

### Characterization of *PqMYB113*

*PqMYB113* contained a 798 bp open reading frame (ORF) encoding 266 amino acids (GenBank accession number: QCF29938.1). The online software ProtParam analysis showed that the theoretical molecular weight of PqMYB113 was approximately 29.90 kD and the theoretical isoelectric point was 8.83. The protein instability coefficient was 55.91, indicating that it was an unstable protein. The maximum hydrophilicity/hydrophobicity of the protein encoded by *PqMYB113* was approximately 1.444, and the minimum was −2.578. The hydrophilic mean (GRAVY) was −0.557, suggesting that the protein was a hydrophilic protein (Supplementary Figure 1A). SignalP 4.1 Server and TMHMM predicted that PqMYB113 did not have a signal peptide site or transmembrane region, indicating that it is a non-secreted protein and non-transmembrane protein (Supplementary Figures 1B,C). Secondary structure prediction showed that PqMYB113 contained 35.09% α- helices, 4.91% β-turns, 10.19% extended chains and 49.81% random coils. The three-dimensional structure was constructed by SWISS-MODEL. The similarity of the model was 60.91% with the transcription factor WER (Supplementary Figure 1D). Conservative domain analysis showed that there were two conserved domains of SANT (repeat R) at the N-terminus, which revealed that PqMYB113 belonged to the 2R-MYB subfamily.

### Phylogenetic Analysis and Sequence Alignment of PqMYB113

**FIGURE 1:**
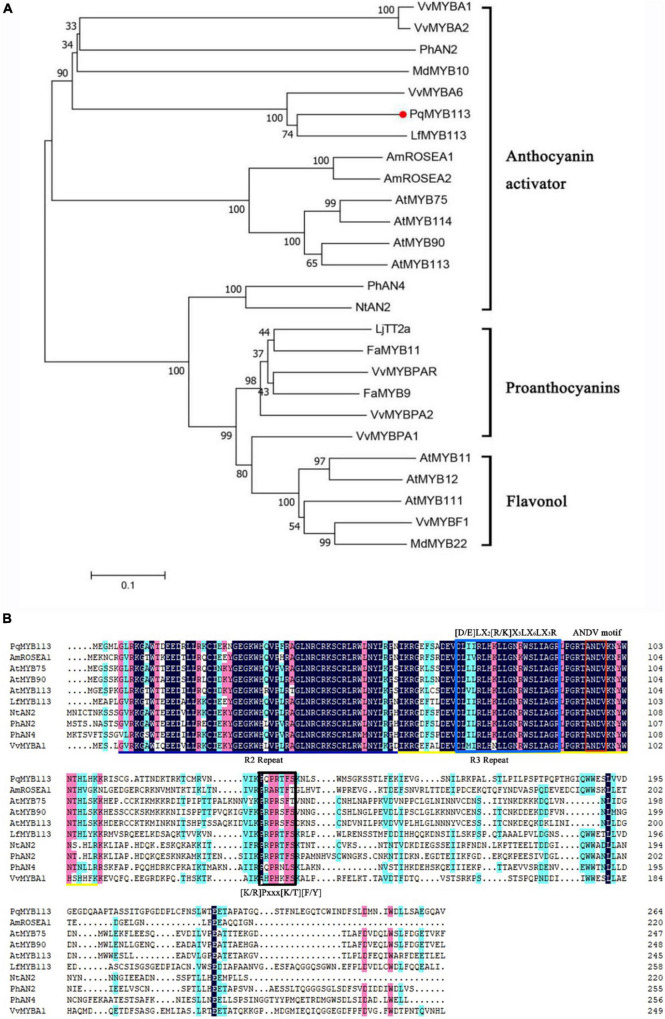
Phylogenetic analysis and sequence alignment of PqMYB113. **(A)** The phylogenetic analysis of PqMYB113 protein and MYB proteins from other species. PqMYB113 is highlighted with a red dot. The neighbor-joining method with MEGA software was used to construct the phylogenetic tree. Bootstrap values as a percentage of 1000 replicates are indicated at corresponding branch nodes. Putative functions of the R2R3-MYBs are listed on the right. **(B)** The alignment of deduced amino acid sequences of *PqMYB113* with other similar R2R3-MYB proteins. Alignment was conducted using DNAMAN Version 8. The R2 (black line) and R3 (yellow line) MYB domains shown refer to two repeats of the MYB DNA-binding domain of MYB proteins. The blue box shows the motif that interacts with bHLHs. The red and black boxes show the ANDV motif and motif 6, respectively.

Phylogenetic analysis showed that PqMYB113 was relatively far from the MYB transcription factors that regulate the synthesis of proanthocyanidins, such as grape VvMYBPA2, strawberry FaMYB9 and FaMYB11, and the synthesis of flavonols, such as *Arabidopsis* AtMYB11 and grape VvMYBF1 (Figure 1A). It was clustered with the MYB transcription factor that promotes anthocyanin synthesis in other plants, such as *Liquidambar* LfMYB113, *Arabidopsis* AtMYB75/90/113/114, grape VvMYBA1, VvMYBA6, apple MdMYB10, and petunia PhAN2 (Figure 1A), suggesting that PqMYB113 probably has the same function as these transcription factors.

Sequence analysis revealed that PqMYB113 contained R2 and R3 MYB DNA-binding domains in the N-terminus, indicating that PqMYB113 was an R2R3-MYB transcription factor (Figure 1B). The [D/E]LX2[R/K]X3LX6LX3R motif, which is responsible for interacting with bHLH proteins, was found in the R3 domain of PqMYB113 (Figure 1B; Zimmermann et al., 2004), suggesting that PqMYB113 could interact with bHLH. ANDV and KPRPR[S/T]F motifs, which are characteristics of anthocyanin biosynthesis regulators, were also found in PqMYB113 (Figure 1B; Yamagishi et al., 2010), suggesting that PqMYB113 is probably involved in the regulation of anthocyanin synthesis.

### Expression Analysis of *PqMYB113* and Anthocyanin Biosynthetic Genes in the Leaves of *P. qiui*

**FIGURE 2:**
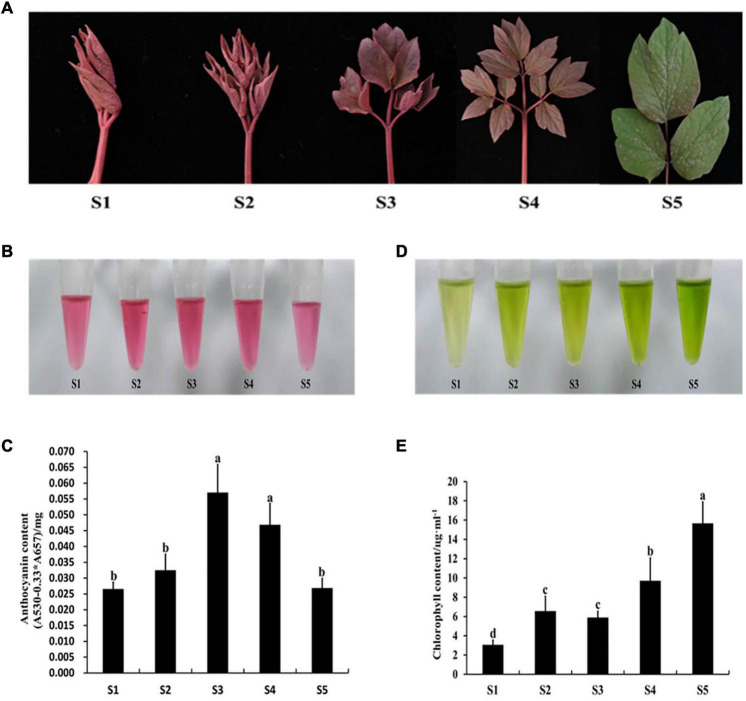
Pigment content at different stages in *P. qiui.* **(A)** The leaves phenotype in different stages of *P. qiui*. **(B)** The anthocyanin extracting solution colors in leaf. **(C)** The anthocyanin content in leaf. **(D)** The chlorophyll extracting solution colors in leaf. **(E)** The chlorophyll content in leaf. S1, leaves of 10 days after sprouting; S2, leaves of 20 days after sprouting; S3, leaves of 30 days after sprouting; S4, leaves of 40 days after sprouting; S5, leaves of 50 days after sprouting. a, b, c, and d indicate significant difference at *p* ≤ 0.05 level by Duncan’s test.

**FIGURE 3:**
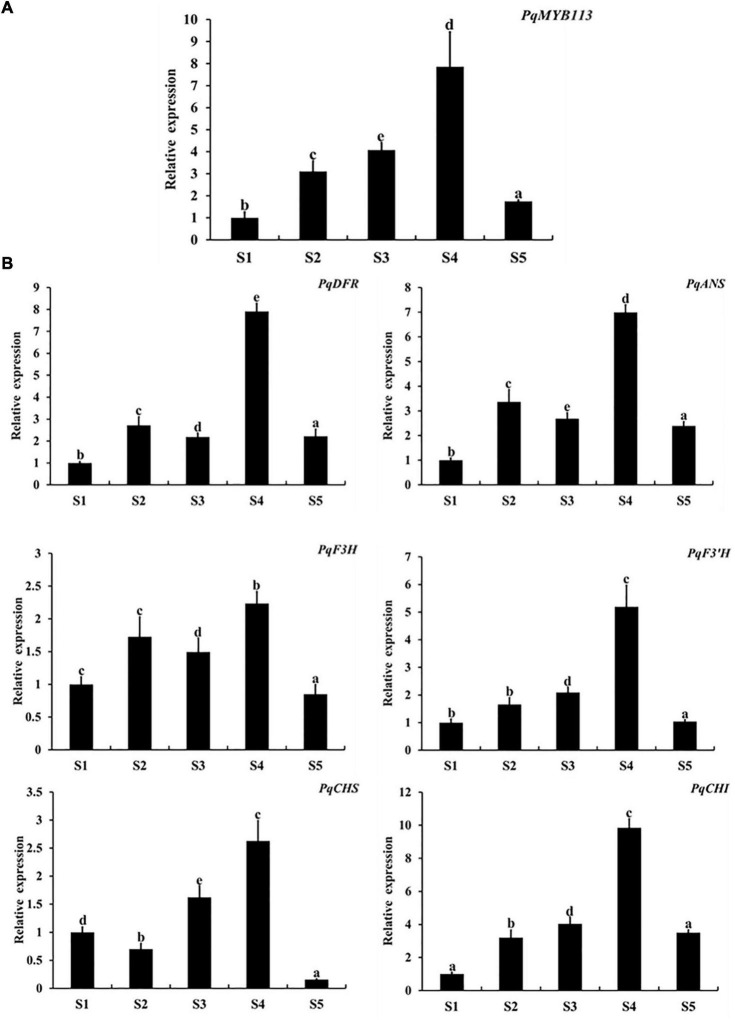
Expression level of anthocyanin structural genes and *MYB113* at different stages in *P. qiui.* **(A)** The expression level of *PqMYB113* at different stages. **(B)** The expression level of anthocyanin structural genes at different stages. S1, leaves of 10 days after sprouting; S2, leaves of 20 days after sprouting; S3, leaves of 30 days after sprouting; S4, leaves of 40 days after sprouting; S5, leaves of 50 days after sprouting. Ubiquitin was used as an internal control. Error bars represent the standard errors. a, b, c, d, and e indicate significant differences at the *p* ≤ 0.05 level by Duncan’s test.

The expression levels of *PqMYB113* and anthocyanin biosynthetic genes in different leaf stages were revealed by qRT-PCR, and the anthocyanin content and chlorophyll content were measured with a UV spectrophotometer (Figure 2). The results showed that the anthocyanin content in leaves at S3 was the highest, followed by S4, the lowest at S1 and S5 (Figures 2B,C). Chlorophyll showed an increasing trend from S1 to S5 (Figures 2D,E). The expression level of *PqMYB113* was the highest at S4, followed by S2 and S3, and the lowest at S1, which was positively correlated with the anthocyanin content in leaves (Figures 3A, 2C). In addition to *PqMYB113*, the anthocyanin pathway genes *PqCHS*, *PqDFR*, *PqANS, PqF3H, PqF3′H*, and *PqCHI* presented a basically consistent trend with the anthocyanin content in leaves (Figure 3B).

### PqMYB113 Was a Transcriptional Activator

**FIGURE 4:**
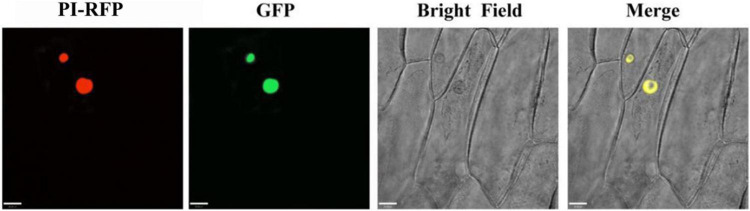
The subcellular localization of GFP fusion of PqMYB113. Onion epidermal cells transiently expressing GFP and PqMYB113-GFP under the control of the CaMV 35S promoter. PI-RFP, mCherry fluorescence; GFP, GFP fluorescence; Merge, merged images of mCherry fluorescence; GFP fluorescence and bright-field microscopy. Bars = 33 μm.

To investigate the subcellular localization of PqMYB113, the recombinant vector pCAMBIA1301-PqMYB113-GFP was transformed into onion cells and observed with confocal laser scanning microscopy. Onion cells expressing the PqMYB113-GFP fusion protein displayed a strong signal in the cell nucleus. The results revealed that PqMYB113 was localized in the cell nucleus (Figure 4).

**FIGURE 5:**
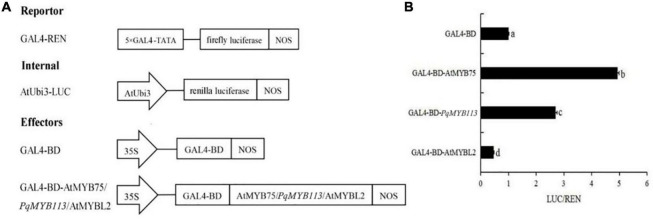
The Dual Luciferase Transient Transfection Assay of *PqMYB113*. **(A)** The schematic depiction of the constructs used in the *Arabidopsis* protoplast co-transfection assay. **(B)** The relative luciferase activity (LUC/REN). a, b, c, and d indicate significant differences at the *p* ≤ 0.05 level by Duncan’s test.

To examine the transcriptional activity of PqMYB113, transient reporter assays were used (Figure 5). AtMYB75 was used as a positive control, and AtMYBL2 was used as a negative control. As shown in Figure 5, the relative luciferase activity was increased nearly three times by the expression of *GAL-BD-AtMYB113* compared with the *GAL4-BD* control. This result suggested that PqMYB113 was a transcriptional activator.

### Overexpression of *PqMYB113* in *Arabidopsis* and Tobacco Promoted Anthocyanin Accumulation and the Expression of Anthocyanin Pathway Genes

**FIGURE 6:**
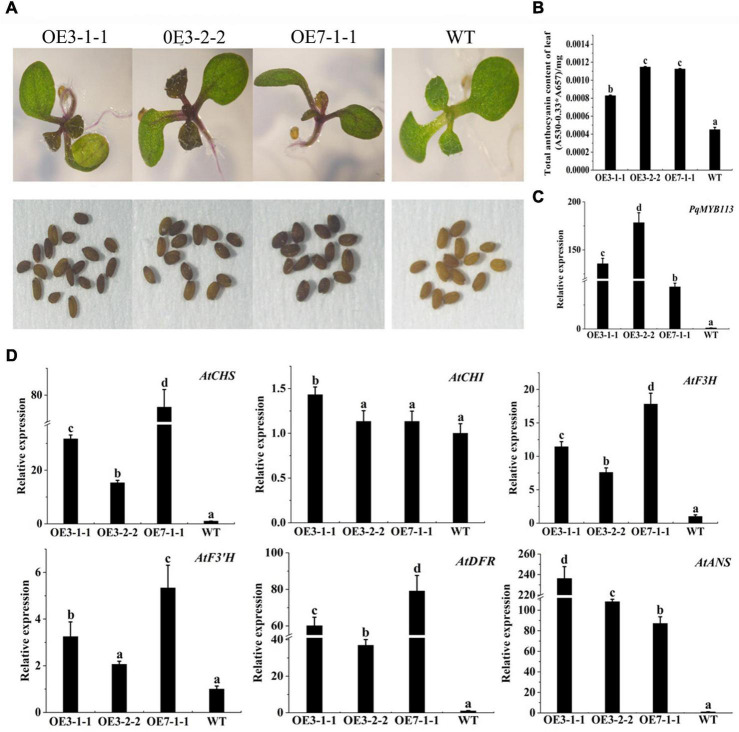
The phenotype and the effect of *PqMYB113* overexpression in transgenic *Arabidopsis* plants. **(A)** The seeding and seeds phenotype of transgenic overexpression lines OE 3-1-1, OE 3-2-1, and OE 7-1-1 in comparison to WT. *Arabidopsis* seedlings were grown on MS plates at 22°C for 10 days. **(B)** The anthocyanin content in leaves. **(C)** The expression level of *PqMYB113* gene in leaves. **(D)** The expression level of anthocyanin biosynthesis structure genes in leaves. a, b, c, and d indicate significant differences at the *P* ≤ 0.05 level by Duncan’s test.

**FIGURE 7:**
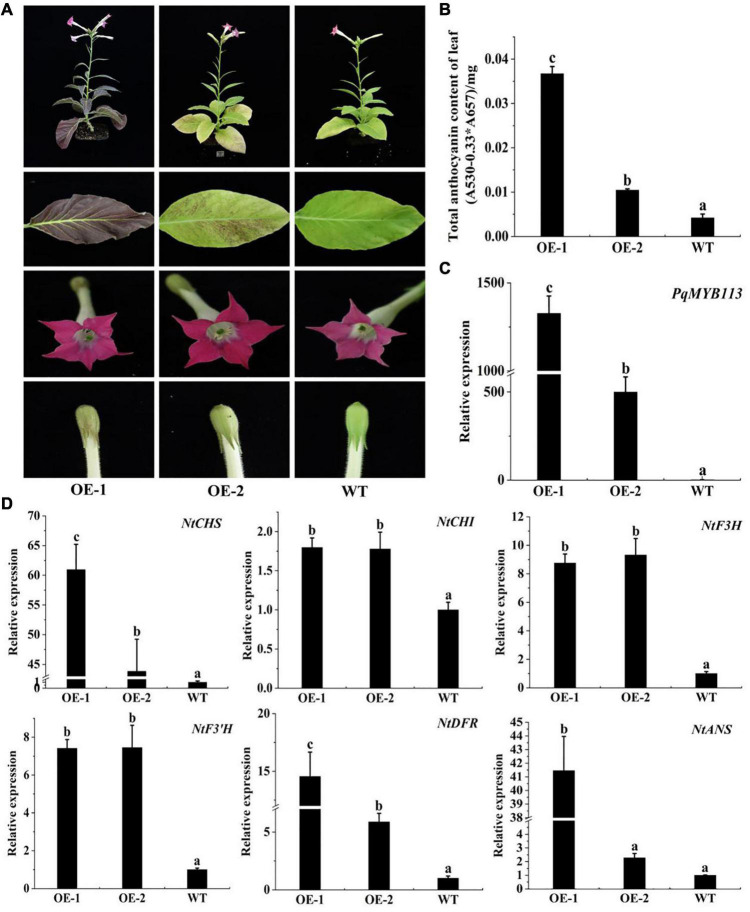
The phenotype and the effect of *PqMYB113* overexpression in transgenic tobacco. **(A)**. The phenotype of OE-1 and OE-2 in comparison to WT. **(B)** The anthocyanin content in leaves. **(C)** The expression level of the *PqMYB113* gene in leaves. **(D)** The expression level of anthocyanin biosynthesis structure genes. a, b, and c indicate significant differences at the *P* ≤ 0.05 level by Duncan’s test.

To characterize the function of *PqMYB113*, the coding sequence of *PqMYB113*, driven by the 35S promoter, was transformed into *Arabidopsis* and tobacco. In *Arabidopsis*, three T3 overexpression (OE) transgenic lines, designated as OE 3-1-1, OE 3-2-2, and OE 7-1-1, were generated. Compared with the wild-type (WT), all *PqMYB113* OE lines showed increased anthocyanin pigments in young leaves (Figures 6A,B). In addition, the seed coat color of transgenic plants was also deeper than that of WT (Figure 6A), suggesting more proanthocyanidin accumulation in the seed coat (Hui et al., 2019). In transgenic tobacco, the leaves of *PqMYB113* OE-1 lines were dark red, and the leaves of OE-2 plants had red spots (Figure 7A). The color of the petals and sepals of the OE lines was slightly redder than that of WT (Figure 7A). In *PqMYB113* OE *Arabidopsis* plants, the expression levels of anthocyanin biosynthesis pathway genes were analyzed with qRT-PCR. Compared with WT, the *PqMYB113* gene was highly expressed in the transgenic plants, and there was little or no expression in wild-type *Arabidopsis* (Figure 6C). The expression of *AtCHS, AtF3H*, *AtF3′H, AtDFR*, and *AtANS* in transgenic lines was higher than that of WT, especially *AtCHS, AtDFR*, and *AtANS.* The expression of *AtCHI* in *Arabidopsis* overexpression plants was similar to that in WT plants (Figure 6D). In *PqMYB113* OE tobacco, the results were similar to those in *Arabidopsis* (Figure 7). All of these results suggested that *PqMYB113* can promote anthocyanin accumulation by regulating anthocyanin pathway genes.

### PqMYB113 Activates the Promoters of Anthocyanin Synthesis Pathway Genes

**FIGURE 8:**
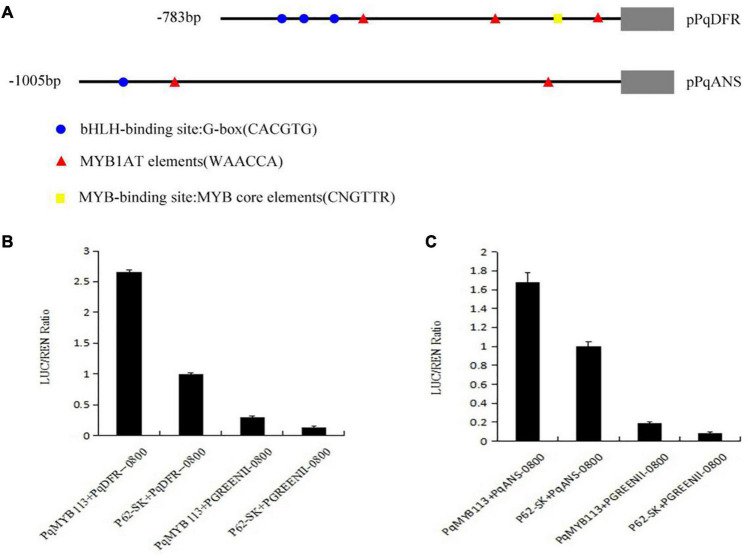
Dual luciferase transient expression assay of *PqDFR, PqANS* promoters and PqMYB113. **(A)** The distribution of MYB binding and bHLH-binding elements in the promoter sequences of *PqDFR* and *PqANS*. **(B)** The effect of *PqDFR* promoter activity by PqMYB113. **(C)** The effect of *PqANS* promoter activity by PqMYB113. The ratio of LUC/REN of the empty vector (SK) plus promoter was used as calibrator (set as 1).

To test the effect of PqMYB113 on the key anthocyanin structural genes, a dual luciferase reporter assay was carried out. Among the anthocyanin pathway genes, *DFR* and *ANS* are the key structural genes (Zhong et al., 2020). Therefore, the promoters of *PqDFR* and *PqANS* were chosen as potential targets of PqMYB113 transcription activation. The promoter lengths of *PqDFR* and *PqANS* were 783 bp and 1005 bp, respectively. The key elements of the promoters were analyzed by PlantCARE online software, and it was found that these two promoters both have MYB binding sites (Figure 8A), suggesting that MYB may regulate their activity. As shown in Figures 8B,C, the promoter activities of *PqDFR* and *PqANS* increased approximately 2.6- and 1.7-fold by PqMYB113, respectively. The results suggested that PqMYB113 can activate the promoters of *PqDFR* and *PqANS*.

### PqMYB113 Can Interact With Basic Helix Loop Helix and WD40

Bimolecular fluorescence complementation (BiFC) assays and yeast two-hybrid systems were performed to investigate whether PqMYB113 interacts with bHLH and WD40. In this study, PqbHLH1 and PqWD40 were used as candidate co-activators based on our previous study (Luo et al., 2017; Supplementary Figure 2).

**FIGURE 9:**
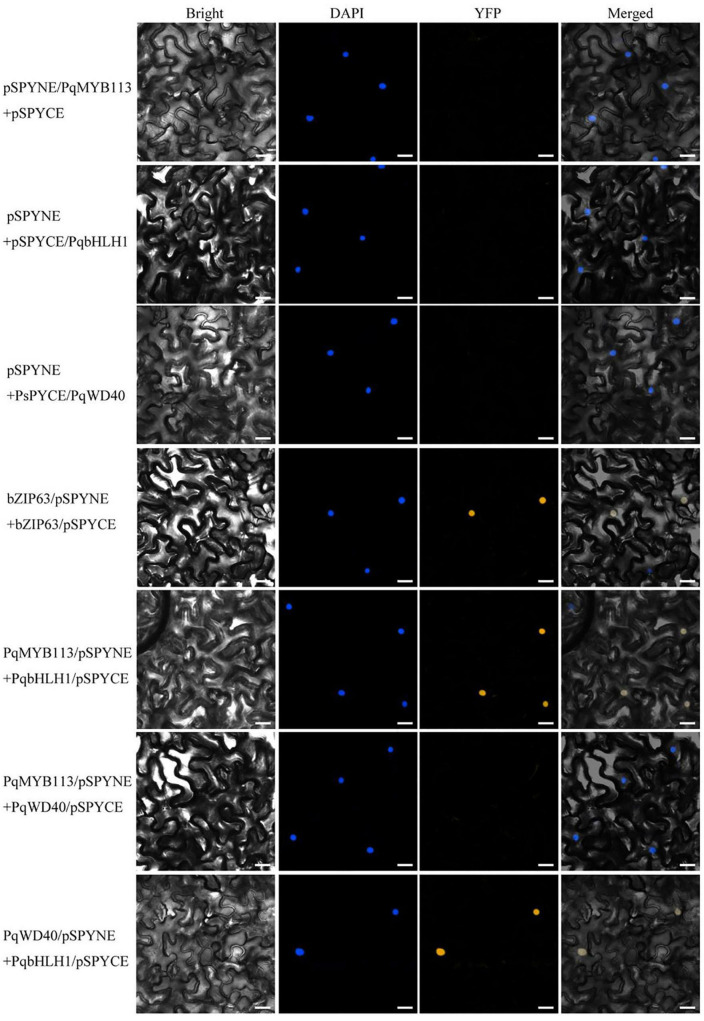
Bimolecular fluorescence complementation in *Nicotiana benthamiana* leaves. Bright, field images show the complete epidermal cell profile in bright-field view; YFP, fluorescence of YFP; DAPI, nuclear staining; “Merge” is merged with chloroplast autofluorescence, YFP fluorescence and bright field images. Bars = 25 μm.

In BiFC, pSPYNE/bZIP63 + pSPYCE/bZIP63 was used as a positive control, and pSPYNE/PqMYB113 + pSPYCE, pSPYNE/PqbHLH1 + pSPYCE, pSPYNE/PqbWD40 + pSPYCE were used as negative controls. As shown in Figure 9, a yellow fluorescent protein (YFP) fluorescence signal was observed in the nuclei when pSPYNE/PqMYB113 was co-expressed with pSPYCE/PqbHLH1 or pSPYNE/PqbHLH1 with pSPYCE/PqbWD40 but not when pSPYNE/PqMYB113 was co-expressed with pSPYCE/PqbWD40 or any negative controls. The results suggested that PqMYB113 could interact with PqbHLH1 but not with PqWD40, and PqbHLH1 could interact with PqWD40.

**FIGURE 10:**
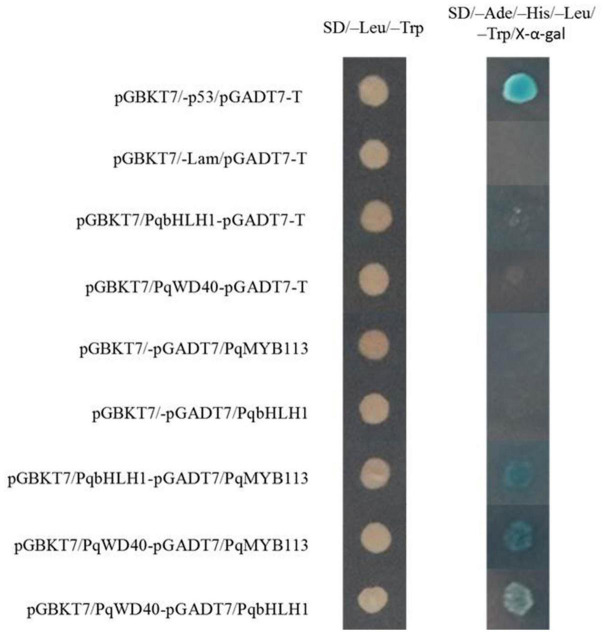
The interaction between PqMYB113 and PqbHLH1, PqWD40 in the yeast two-hybrid system. pGBKT7-53/pGADT7-T served as a positive control; pGBKT7/-Lam/pGADT7-T served as a negative control.

In the yeast two-hybrid system, yeast colonies expressing pGBKT7-53 and pGADT7-T (positive control), PqMYB113 and PqbHLH1, PqbHLH1 and PqWD40, PqMYB113 and PqWD40 grew well on SD/-Leu/-Trp medium and became blue on SD/-Ade/-His/-Leu/-Trp/X-α-Gal medium (Figure 10). These results also indicated that PqMYB113 could interact with PqbHLH1 and PqWD40.

## Discussion

Increasing numbers of studies indicate that R2R3-MYB TFs are important regulators of anthocyanin biosynthesis in many plants (Liu et al., 2015). However, how R2R3-MYB TFs regulate anthocyanin biosynthesis is not clear in tree peony leaves. Here, an R2R3-MYB TF (PqMYB113) was isolated from *P. qiui*. The deduced amino acid sequence analysis of PqMYB113 revealed the presence of the highly conserved R2 and R3 domains at the N-terminus, which is characteristic of R2R3-MYB transcription factors (Figure 1B), indicating that PqMYB113 was an R2R3-MYB transcription factor. The characteristic motifs of anthocyanin biosynthesis regulators (ANDV and KPRPR[S/T]F) were also found in PqMYB113 (Figure 1B). Phylogenetic analysis showed that PqMYB113 belongs to subgroup 6 (SG6) and clustered with other anthocyanin MYB regulators together into the anthocyanin clade (Figure 1A). PqMYB113 shared the highest identity with *Liquidambar formosana* LfMYB113 (58.09%), followed by grapevine VvMYBA1 (48.87%), petunia PhAN2 (47.99%), and *Arabidopsis* AtMYB90 (44.36%) (Figure 1B). PqMYB113 showed the lowest identity with *Antirrhinum majus* AmROSEA1 (39.71%) (Figure 1B). Subcellular localization analysis revealed that PqMYB113 was localized in the cell nucleus (Figure 4). The luciferase assay revealed that PqMYB113 was a transcriptional activator (Figure 5). In addition, the [D/E]LX2[R/K]X3LX6LX3R motif, which is responsible for interacting with bHLH proteins, was found in the R3 domain of PqMYB113 (Figure 1B; Zimmermann et al., 2004). Taken together, these results suggested that PqMYB113 probably functions as an R2R3-MYB transcription activator that is dependent on bHLH proteins in regulating anthocyanin biosynthesis.

The anthocyanin biosynthesis MYB regulator can promote the accumulation of anthocyanin by regulating the expression level of flavonoid biosynthesis pathway genes. In apple skin, MdMYB3 can regulate anthocyanin biosynthesis and promote anthocyanin accumulation. Overexpression of *MdMYB3* in tobacco up-regulated the anthocyanin content and the expression levels of *NtCHS*, *NtCHI*, and *NtUFGT* (Vimolmangkang et al., 2013). In grape hyacinth, the expression level of *MaAN2* was positively correlated with the anthocyanin content and the expression level of *MaDFR* and *MaANS*; meanwhile, MaAN2 can also strongly activate the promoters of *MaDFR* and *MaANS* (Chen et al., 2017). Here, the expression of *PqMYB113* decreased gradually during tree peony leaf development, and it was positively correlated with anthocyanin accumulation and the expression profiles of *PqCHS*, *PqDFR*, and *PqANS*. In the transgenic *Arabidopsis* plants, compared with *AtF3H* and *AtF3′H*, the expression levels of *AtCHS*, *AtDFR* and *AtANS* in transgenic lines were much higher than those of the wild-type (Figure 6). Similar results were observed in transgenic tobacco (Figure 7). All of these results suggested that *PqCHS*, *PqDFR*, and *PqANS* (especially *PqDFR* and *PqANS*) may be the key structural genes of anthocyanin synthesis in tree peony leaves, and that PqMYB113 could activate these genes, which was also confirmed by a dual luciferase reporter assay (Figure 8).

In apple, overexpression of *MdbHLH3* independently promoted red fruit coloration, and this coloration was enhanced by low temperature. *MdbHLH3* suppression inhibited red coloration in the skin around the infiltration site, while the empty vector control did not influence red coloration (Xie et al., 2012). In the present study, overexpression of *PqMYB113* in tobacco and *Arabidopsis* plants promoted the accumulation of anthocyanin (Figures 6, 7). However, overexpression of *PqbHLH1* did not promote anthocyanin accumulation (data not shown). This may be because the R2R3-MYB TFs play crucial roles in anthocyanin biosynthesis and are usually considered more specific in the MBW complex, while bHLH may function as an enhancer to promote the transcriptional activation activity of MYB. Bimolecular fluorescence complementation assays showed that PqMYB113 can interact with PqbHLH1 but not with PqWD40, and PqbHLH1 can interact with PqWD40 (Figure 9). Similar results were also found in apple and tomato (An et al., 2012; Gao et al., 2018). In apple, MdTTG1 (WD40) interacted with bHLH transcription factors but not MYB protein, whereas bHLH was known to interact with MYB (An et al., 2012). In tomato, SlAN11 (WD40) interacted with bHLH but not with MYB proteins in the ternary MBW complex, whereas bHLH interacted with MYB (Gao et al., 2018). The possible reason was that in addition to enhancing the activity of MYB, bHLH can bridge the MYB and WD40 proteins, while the function of WD40 was to stabilize the bHLH-MYB transcriptional complex, thus allowing the binding of the resulting MBW complex to promoters of anthocyanin pathway genes (*DFR*, *ANS*, etc.).

## Conclusion

PqMYB113 is an R2R3-MYB transcription factor that has an ANDV motif and motif 6, which are characteristics of anthocyanin biosynthesis activators. Its expression is positively correlated with anthocyanin accumulation in tree peony leaves. PqMYB113 could form a ternary MBW complex with PqbHLH1 and PqWD40 and promoted anthocyanin accumulation by up-regulating the expression levels of *PqDFR* and *PqANS*.

## Data Availability Statement

The original contributions presented in the study are included in the article/Supplementary Material, further inquiries can be directed to the corresponding author/s.

## Author Contributions

JL and YZ conceived and designed the research. XL, JD, DH, QL, and QW conducted the experiments and analyzed the data. XL and JD wrote the manuscript. JL and LN modified the manuscript. All authors have read and agreed to the manuscript.

## Conflict of Interest

The authors declare that the research was conducted in the absence of any commercial or financial relationships that could be construed as a potential conflict of interest.

## Publisher’s Note

All claims expressed in this article are solely those of the authors and do not necessarily represent those of their affiliated organizations, or those of the publisher, the editors and the reviewers. Any product that may be evaluated in this article, or claim that may be made by its manufacturer, is not guaranteed or endorsed by the publisher.

## Footnotes

1
https://www.ncbi.nlm.nih.gov/orffinder/
