# Supplementary material for: The Paeonia qiui R2R3-MYB Transcription Factor PqMYB113 Positively Regulates Anthocyanin Accumulation in Arabidopsis thaliana and Tobacco
Source: Front Plant Sci. 2022 Jan 12;12:810990. doi: 10.3389/fpls.2021.810990 (PMC8789887; doi:10.3389/fpls.2021.810990)
Supplement: Supplementary file 1 [file Data_Sheet_1.docx]

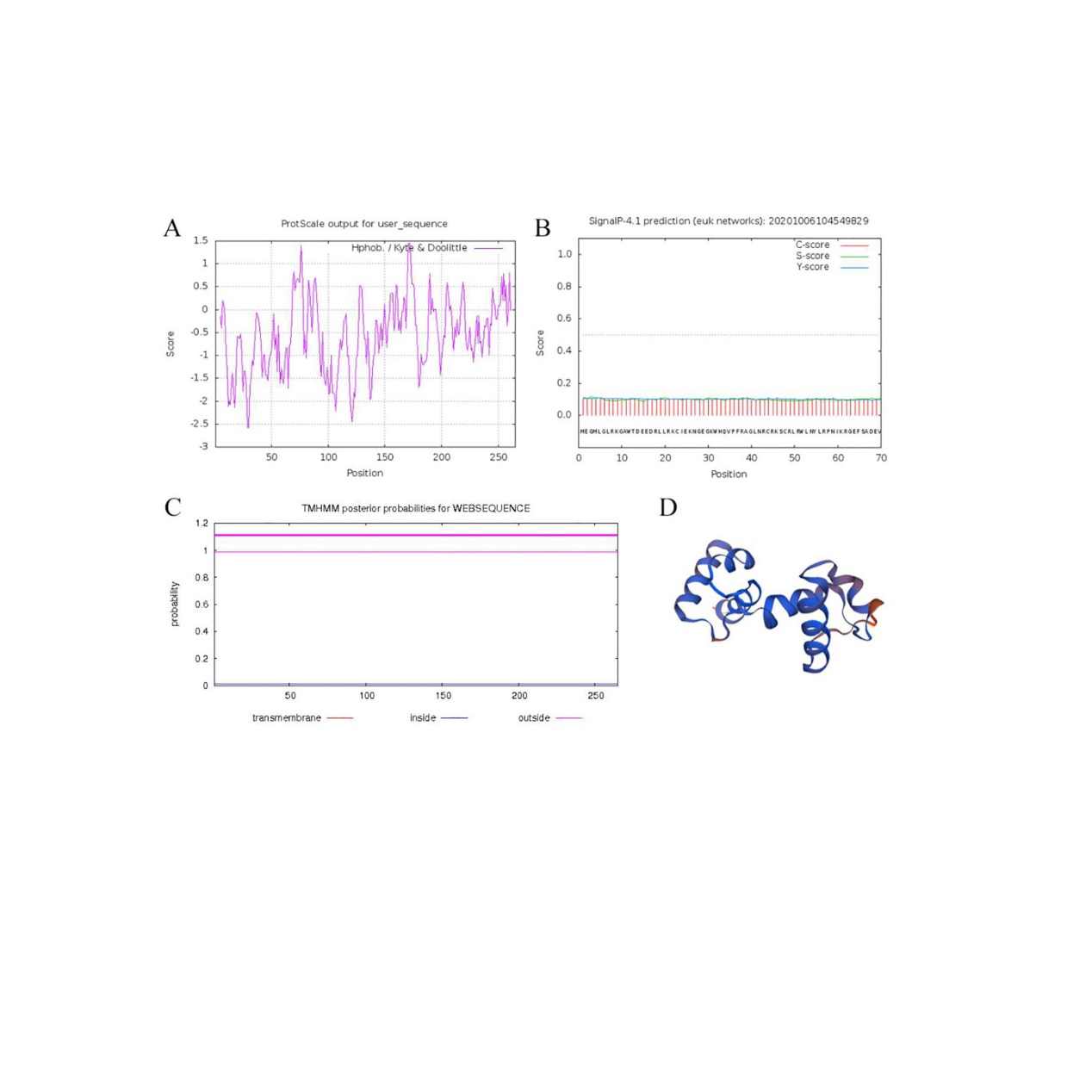


**Figure S1.** The bioinformatics analysis of PqMYB113 protein. **(A)** hydrophilic and hydrophobic analysis; **(B)** signal peptide analysis; **(C)** transmembrane analysis; **(D)** tertiary structure prediction.


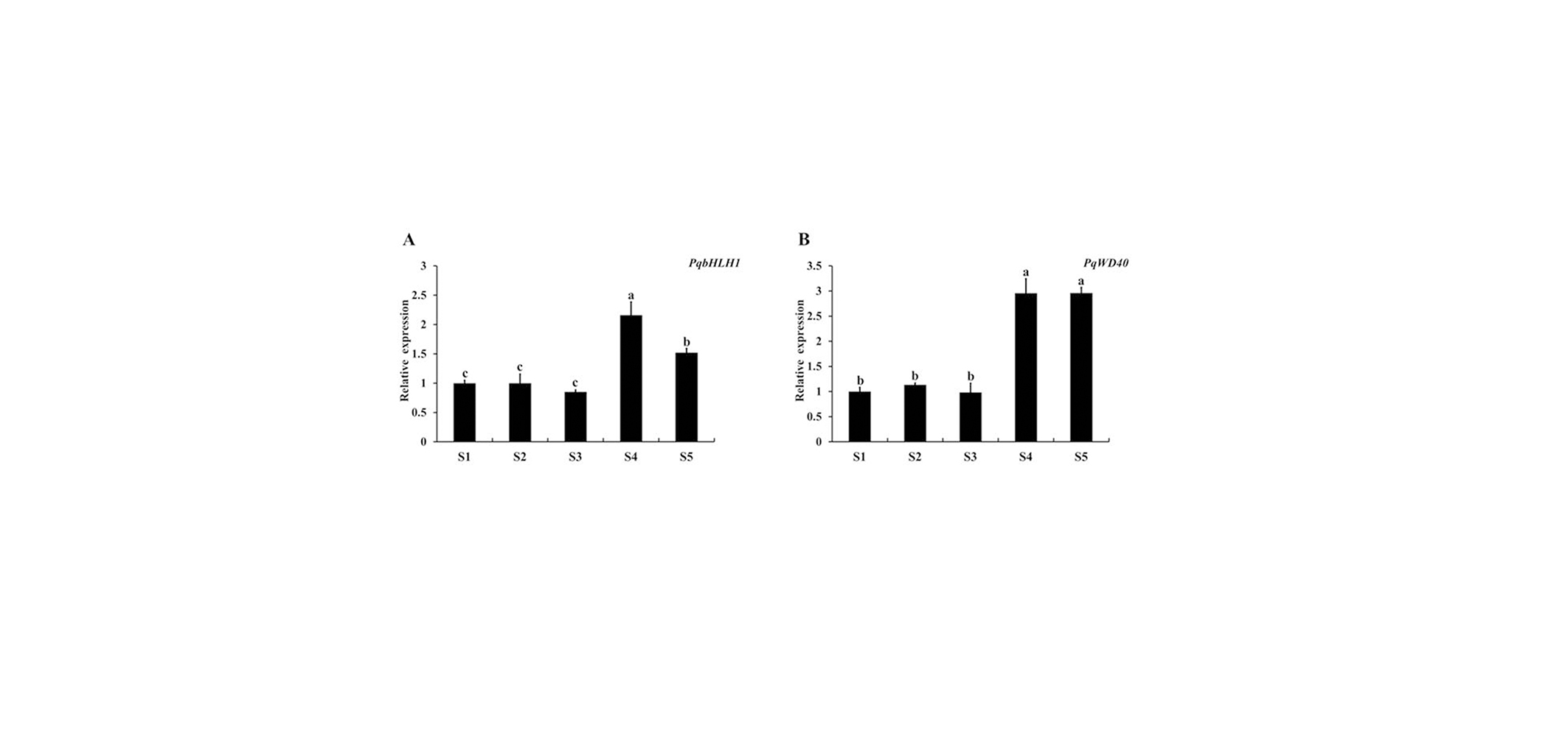


**Figure S2.** Expression level of *bHLH1*and *WD40* at different stages in *P. qiui.* (A) Expression level of *PqbHLH1* at different stages. (B) Expression level of *PqbHLH1* at different stages.a, b and c indicate significant differences at the p ≤ 0.05 level by Duncan’s test.

**Table S1** The gene specific primers used for qRT-PCR analysis

| Primer name | Forward primer | Reverse primer |
| --- | --- | --- |
| *PqMYB113* | GCTGTAGATTGAGGTGGTT | TCTTCTTGTGTAGGTGTGTGT |
| *PqUbiquitin* | GACCTATACCAAGCCGAAG | CGTTCCAGCACCACAATC |
| *PqMYB113* | GCTGTAGATTGAGGTGGTTGA | TCTTCTTGTGTAGGTGTGTGT |
| *PqCHS* | CTGCTATCATCATTGGTTCGG | GTCGCTTGTAGTTTTTCGGGT |
| *PqCHI* | CTATTCTTTTCACACAGACACC | TGCTTCCCTATGATCGACTCC |
| *PqF3H* | CGAAATCCCAATCATCTCCCT | TATTTCACGCCAATCTCGCAC |
| *PqF3’H* | TGATGTTGATGGAGAGGGTGG | GCTAGGATTTTAGGGTGTCGG |
| *PqDFR* | GAGAATATGAGGAAGGTGAAG | ACACGAAATACATCCATCCAG |
| *PqANS* | ACCAGCATCACCAACATCTTC | GCATATTTCTCCTTCTCTTCC |
| *AtActin* | AACCACTATGTTCTCAGGTATCGCT | TGGACCTGCCTCATCATACTCG |
| *AtCHS* | GCATCTTGGCTATTGGCACTG | CGTTTCCGAATTGTCGACTTGT |
| *AtCHI* | CTCCTCCAATCCATTATTCCTCG | TTTCCCTTCCACTTGACAGATAGAG |
| *AtF3H* | GTGTTTAGCGACGAAATCCCG | ACGAGCGAGACGAGTCATATCC |
| *AtF3'H* | TCGTGGTCGCCGCTTCTAA | CCATCGGTGTCCGTAAGGTG |
| *AtDFR* | CAAACGCCAAGACGCTACTCA | CATTCACTGTCGGCTTTATCACTTC |
| *AtANS* | ACGGTCCTCAAGTTCCCACAA | CAGCTCCTCAATACAATTCTCACG |
| *AtFLS* | CTGAGGTTGAGTAATGGGAGG | TTGCGGTAACTGTAATCCTTG |
| *NtActin* | ATGAGTTGCGTGTTGCTCCT | TACCTGTTGTACGGCCACTG |
| *NtCHS* | TGACACCCACTTGGATAGTTTAG | CGACCTCTGGAATTGGATCAG |
| *NtCHI* | GGATGATGGTGCGGTTGGTA | GTCATCGGATGCTTCACCCA |
| *NtF3H* | CCGACCTTACCCTTGGACTG | TCGAGTTCACCACTGCTTGA |
| *NtF3’H* | AGGCTCAACACTTCTCGT | CATCAACTTTGGGCTTCT |
| *NtDFR* | AACCAACAGTCAGGGGAATG | TTGGACATCGACAGTTCCAG |
| *NtANS* | TGGCGTTGAAGCTCATACTG | GGAATTAGGCACACACTTTGC |

**Table S2** The primers for cloning promoter of *PqDFR* and *PqANS*

| Primer name | Primer sequence |
| --- | --- |
| PqDFR-SP1 | TCCACAAGGTCAAATGGGTATC |
| PqDFR-SP2 | AAAAATAAAACCACCCCCACAG |
| PqDFR-SP3 | GACGTAGCCATGTTCCAGAAG |
| PqANS-SP1 | CATTTCTCCCTAAGCTTGATGTCC |
| PqANS-SP2 | GCCCTTCTTCTTTCTTCTCTTCCTC |
| PqANS-SP3 | CGCACGTATTCTTTTGGGATGG |

**Table S3** The primers used for constructing vectors of dual luciferase assay

| Primer name | Primer sequence |
| --- | --- |
| PqMYB113-62-F | GGCCGCTCTAGAACTAGTGGATCCATGGAGGGAATGTTAGGATTG |
| PqMYB113-62-R | ATCGATAAGCTTGATATCGAATTCTTAATTCACCGCTTGCCCTTC |
| PqDFR-0800-F | GTACCGGGCCCCCCCTCGAGGTCGACAGGCTGCACGCAAGGAATTA |
| PqDFR-0800-R | GGCTGCAGGAATTCGATATCAAGCTTTTGCTTTTGTTTTTTAACCAC |
| PqANS-0800-F | GTACCGGGCCCCCCCTCGAGGTCGACAGAGGAGAAGCATAGGCATT |
| PqANS-0800-R | GGCTGCAGGAATTCGATATCAAGCTTTTTTGCAGCAACGTTTACTC |

**Table S4** The genes used in this study and their accession numbers

| Species | Gene | Accession Number |
| --- | --- | --- |
| *Anthurium andraeanum* | AmROSEA1 | ABB83826 |
| *Anthurium andraeanum* | AmROSEA2 | DQ275530.1 |
| *Arabidopsis thaliana* | AtMYB11 | AEE80369.1 |
| *Arabidopsis thaliana* | AtMYB12 | AEC10843.1 |
| *Arabidopsis thaliana* | AtMYB75 | AF062908 |
| *Arabidopsis thaliana* | AtMYB90 | AF062915 |
| *Arabidopsis thaliana* | AtMYB111 | AED95797.1 |
| *Arabidopsis thaliana* | AtMYB113 | AY008378 |
| *Arabidopsis thaliana* | AtMYB114 | AY008379 |
| *Fragaria x ananassa* | FaMYB9 | JQ989281.1 |
| *Fragaria x ananassa* | FaMYB11 | JQ989282.1 |
| *Liquidambar formosana* | LfMYB113 | AQM49950.1 |
| *Lotus japonicus* | LjTT2a | AB300033 |
| *Malus domestica* | MdMYB10 | ABB84753 |
| *Malus domestica* | MdMYB22 | AAZ20438.1 |
| *Nicotiana tabacum* | NtAN2 | ACO52472 |
| *Petunia hybrida* | PhAN2 | BAP28593.1 |
| *Petunia hybrida* | PhAN4 | HQ428105.1 |
| *Vitis vinifera* | VvMYBA1 | BAD18977 |
| *Vitis vinifera* | VvMYBA2 | AB097924.1 |
| *Vitis vinifera* | VvMYBA6 | ACL97979.1 |
| *Vitis vinifera* | VvMYBF1 | ACV81697.1 |
| *Vitis vinifera* | VvMYBPA1 | AM259485.1 |
| *Vitis vinifera* | VvMYBPA2 | EU919682.1 |
| *Vitis vinifera* | VvMYBPAR | AB911341 |
| *Vitis vinifera* | VvMYB90 | XP_002274992. |

**Table S5** The primers used for constructing vectors of bimolecular fluorescence complementation assay

| Primer name | Primer sequence |
| --- | --- |
| PqMYB113-N-F | GCCTGGCGCGCCACTAGTGGATCCATGTTAGGATTGAGAAAAGG |
| PqMYB113-N-R | CATCCCGGGAGCGGTACCCTCGAGATTCACCGCTTGCCCTTCAG |
| PqbHLH1 -C-F | GCCTGGCGCGCCACTAGTGGATCCATGACAATGGTTACTGGGATG |
| PqbHLH1 -C-R | CATCCCGGGAGCGGTACCCTCGAGGTTCACACCAGTGATTTTCATTC |
| PqWD40 -N-F | GCCTGGCGCGCCACTAGTGGATCCATGGAGAACTCGACTCAAGA |
| PqWD40-CN-R | CATCCCGGGAGCGGTACCCTCGAGGACCTTCAGAAGCTGCATT |
